# The type of pathogen is associated with organ failure and legacy dysfunction in patients with sepsis

**DOI:** 10.3389/fimmu.2025.1541634

**Published:** 2025-03-31

**Authors:** Qinfen Gao, Zengzheng Li, Jiawei Geng, Bin Han, Tonghua Yang, Shuai Feng, Lijuan Zhao, Yajun Teng, Yafei Li, Renbin Zhao, Wei Zhang, Yi Wang, Yajie Wang

**Affiliations:** ^1^ Department of Emergency, The First People’s Hospital of Yunnan Province, Affiliated Hospital of Kunming University of Science and Technology, Kunming, China; ^2^ Department of Hematology, The First People’s Hospital of Yunnan Province, Affiliated Hospital of Kunming University of Science and Technology, Kunming, China; ^3^ Yunnan Province Clinical Research Center for Hematologic Disease, The First People’s Hospital of Yunnan Province, Kunming, China; ^4^ Yunnan Provincial Clinical Medical Center for Blood Diseases and Thrombosis Prevention and Treatment, The First People’s Hospital of Yunnan Province, Kunming, China; ^5^ Yunnan Atherosclerosis Cooperation Base of Chinese and Western Medicine, The First People’s Hospital of Yunnan Province, Kunming, China; ^6^ Department of Infectious Diseases, The First People’s Hospital of Yunnan Province, Affiliated Hospital of Kunming University of Science and Technology, Kunming, China; ^7^ Department of integrated Chinese and Western medicine, The First People’s Hospital of Yunnan Province, Affiliated Hospital of Kunming University of Science and Technology, Kunming, China; ^8^ Hospital Office, First Affiliated Hospital of Kunming Medical College, Kunming, China

**Keywords:** sepsis, organ dysfunction, pathogenic pathogens, post-sepsis persistent organ dysfunction, survive

## Abstract

**Introduction:**

Is there a difference in pathogen infection among sepsis patients with different organ dysfunction and Post-sepsis persistent organ dysfunction? Is this related to survival? It is currently unclear.

**Methods:**

This study reviewed 1982 sepsis patients between December 2019 and September 2023, and included 619 patients after removing patients with missing data. Of these, 332 were tested for metagenomic next-generation sequencing (mNGS). First, the pathogens distribution was assessed in all NGS-positive patients, followed by patients with different organ dysfunction (excluding those who died during hospitalization). Lastly, the survival analysis was conducted on patients infected with different pathogens.

**Results:**

The results showed that the mortality rate in our cohort was 27.63% in patients with sepsis, and patients with Respiratory, Liver, Circulatory, Hematologic, Neurological, and Renal dysfunction had poor survival. And patients with post-sepsis persistent organ dysfunction after sepsis have worse survival rates. In addition, we found the infection rates of *Legionella* and *Betapapillomavirus* were higher in patients with liver dysfunction. The infection rates of *Mastadenovirus*, *Enterococcus*, and *Candida* were higher in patients with neurological dysfunction. The infection rates of *Candida* were higher in patients with renal dysfunction and hematologic dysfunction. The infection rates of *Moraxella* were higher in patients with circulatory dysfunction. The infection rates of *Enterococcus*, *Pneumocystis*, and *Acinetobacter* were higher in patients with Post-sepsis cardiac dysfunction.The infection rates of Enterococcus, *Acinetobacter*, and Morganella were higher in patients with Post-sepsis liver dysfunction. The infection rates of *Enterococcus*, *Acinetobacter*, and *Staphylococcus* were higher in patients with Post-sepsis respiratory dysfunction. The infection rates of *Enterococcus*, *Candida*, *Pneumocystis*, *Staphylococcus*, and *Listeria* were higher in patients with Post-sepsis renal dysfunction. In addition, we found that patients with *Escherichia* infection in sepsis had the lowest survival rate. The survival rate of patients with *Enterococcus* infection combined with post-sepsis persistent respiratory dysfunction is also worse.

**Discussion:**

In conclusion, there are differences in the types and proportions of pathogens infected in patients with different organ dysfunction and Post-sepsis persistent organ dysfunction. The combination of Escherichia infection and Enterococcus infection with post-sepsis persistent respiratory dysfunction can affect the survival of patients. We should strengthen the management of sepsis patients, especially those with Post-sepsis persistent organ dysfunction.

## Introduction

Sepsis is caused by infections and may result in life-threatening organ dysfunction because of host response disorder (1). The 2016 definition of sepsis (3.0) pays more attention to identifying organ dysfunction in the context of infection (2). Sepsis is the main cause of death in ICU patients globally with increasing incidence rates in developed and developing countries and is related to age, race, comorbidity, and host genetics (3). The complex mechanism of sepsis and its high incidence and mortality rates have increased human health and societal burdens (3). According to statistics, there are more than 18 million cases of severe sepsis worldwide every year, increasing at a rate of 1.5% per year. Furthermore, the fatality rate of sepsis is 28% per annum, with about 14,000 deaths per day globally (4). The most common sources of infection in sepsis patients are *Escherichia* coli, *Staphylococcus aureus*, *Pseudomonas*, *etc.* (5, 6). Timely and accurate identification of pathogens is crucial for improved clinical management and prognosis of these patients (7). Currently, metagenomic next-generation sequencing (mNGS) has the most accurate and sensitive detection methods than other techniques such as blood and sputum cultures (8, 9).

In recent years, with the high attention and standardized diagnosis and treatment of sepsis, its acute mortality rate in the hospital has decreased. It has been estimated that in 2016, 14 million patients suffering from sepsis were hospitalized (10). However, these patients further develop new physical disabilities and cognitive dysfunction, which may further deteriorate their health after discharge. It has been indicated that there is an increased risk of subsequent infection, cardiovascular events, acute renal failure, and inhalation after hospitalization (10). Moreover, Post-sepsis persistent organ dysfunction is related to persistent immune disorders. However, whether pathogens are related to these complications remains undetermined. Therefore, this study employed mNGS to detect pathogens in patients with sepsis to explore whether there is a relationship between infected pathogens and accompanying organ damage and Post-sepsis persistent organ dysfunction. And whether it has an impact on patient survival.

## Methods

### Standards and ethics

A total of 1982 cases of sepsis/septic shock patients diagnosed at the First People’s Hospital of Yunnan Province between December 2019 and September 2023 were reviewed. Patients who lost contact or had missing data were excluded. The definition and diagnostic criteria for sepsis 3.0 jointly released by the American Society for Critical Care Medicine (SCCM) and the European Society for Critical Care Medicine (ESICM) in 2016. The definitions, diagnostic criteria, and requirements formulated by the Poison Conference, that is, sepsis 3.0 = infection + Sequential Organ Failure Assessment (SOFA) ≥ 2 (17, 18). Furthermore, patients who did not meet the criteria for admission or had tumors were excluded, and finally retained 619 patients. The study was approved by the Ethics Committee of the First People’s Hospital of Yunnan Province (No. KHLL2023-KY184) and strictly followed the requirements of the Helsinki Declaration, the International Ethical Code for Human Biomedical Research, the World Health Organization (WHO), and the Council of International Medical Science Organizations in the process of the research.

### Grouping of patients with Post-sepsis persistent organ dysfunction

**Figure 1:**
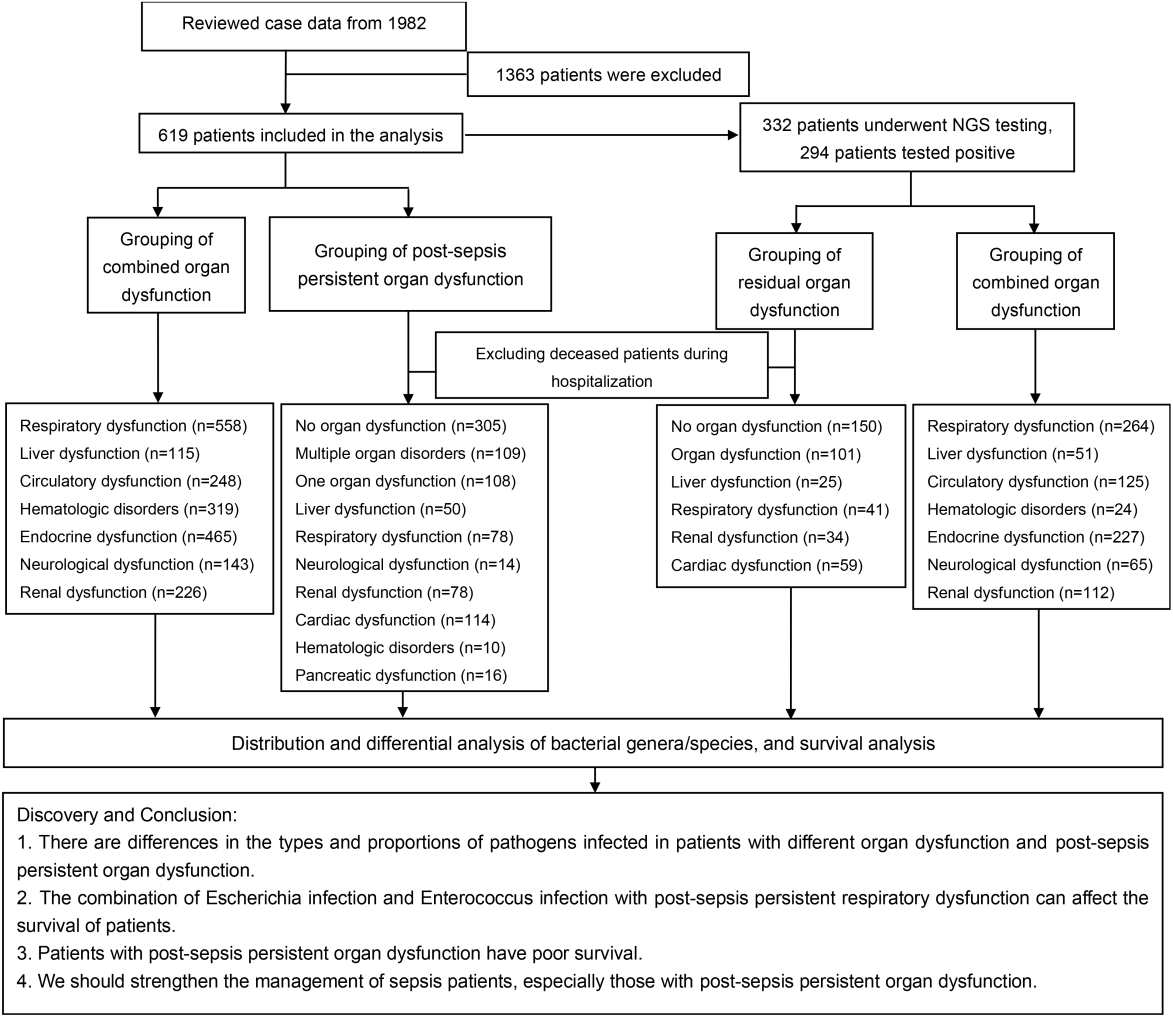
Research diagram.

The cohort of patients who died during the exclusionary hospitalization were followed up by phone on January 30, 2024. Most of the patients went to the hospital for a laboratory examination related to sepsis after discharge. The NGS samples were collected within 1-6 hours of the patient’s admission (before antibiotics treatment) and the results were completed within 24 hours. The samples include (Pleural fluid: 2/332, ascites: 2/332, cerebrospinal fluid: 4/332, alveolar lavage: 22/332, peripheral blood: 302/332). Furthermore, Genskey (China) completed the high-throughput sequencing of infectious pathogens. Patients with Post-sepsis persistent organ dysfunction were grouped according to their examination results and description. That is (10): neurological dysfunction: patients have cognitive dysfunction, limited limb movement, CT examination of brain infarction or ischemic hypoxic encephalopathy, *etc.*, while patients with acute cerebrovascular lesions and other diseases were excluded. Cardiac dysfunction: NT-proBNP ≥ 3000 pg/mL or cTnI ≥ 0.2 ng/mL or cardiac ultrasound EF < 0.5, oral drugs are needed to improve cardiac function. However, individuals with acute myocardial infarction, acute pericarditis, myocarditis, pulmonary embolism, and other diseases were excluded. Liver dysfunction: AST ≥ 200 U/L, ALT ≥ 200 U/L or TBIL ≥ 35 umol/L, and required oral liver-protecting drugs. Additionally, individuals with diseases such as biliary stone obstruction, acute hepatitis, and liver damage caused by poisoning or drugs were excluded. Kidney dysfunction: Cr ≥ 300 umol/L, requiring oral kidney protection drugs or hemodialysis treatment, while excluding patients with drug-induced renal function injury, urinary obstruction, urinary stones, poisoning, and other diseases. Blood system dysfunction: PLT ≤ 50×10^9^/L, requires a dynamic review of blood routine, DIC, and platelet therapy. Moreover, patients with blood systems, immune systems, and hemorrhagic diseases were removed. **Figure 1** shows our research process.

### Statistical method

After exclusion, 619 patients with different organ damage and Post-sepsis persistent organ dysfunction (excluding those who died during hospitalization) were included. Of the 619 patients, 332 were tested for NGS, and 294 were NGS positive. First, the pathogens distribution was assessed in all NGS-positive patients, followed by patients with different organ dysfunction. Lastly, the survival analysis was conducted on patients infected with different pathogens. The SOFA score in this study was expressed by the mean ± standard deviation. Moreover, the mortality rate, the detection frequency of pathogens, and the infection rate of the population were presented by percentage. A chi-square test (IBM SPSS Statistics 21.0) was performed to assess the differences between pathogens, while a Log-rank (Mantel-Cox) test was conducted to evaluate the survival difference. All statistical analyses were carried out and graphs were drawn using GraphPad Prism 9.5.1. *p < 0.05* indicated statistical differences.

## Results

### Patient’s characteristics

**Figure 2:**
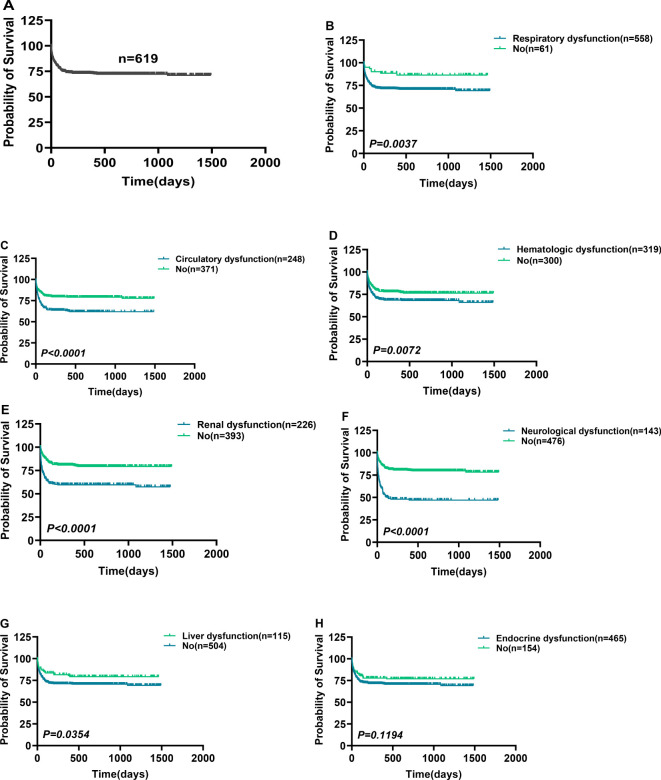
Comparison of survival in patients with different organ dysfunction. Comparison of survival in patients with different organ dysfunction. **(A)** A total of 619 patients were included in this study. **(B–F)** Sepsis-associated respiratory **(B)**, circulatory **(C)**, hematologic **(D)**, renal **(E)**, neurological **(F)**, liver **(G)**, and endocrine dysfunction **(H)** Comparison of survival with non-corresponding dysfunction. *p < 0.0500* = statistically difference, and *p > 0.0500* = no statistical difference.

**Figure 1** shows a brief process.362 patients were excluded from our cohort, and a total of 619 sepsis patients were included, with a mortality rate of 27.63% (**Figure 2A**). Based on the SOFA scores, patients were grouped according to their dysfunction, including liver dysfunction, respiratory dysfunction, endocrine dysfunction, neurological dysfunction, renal dysfunction, Hematologic dysfunction, and Circulatory dysfunction. **Supplementary Table S4** shows the basic characteristics of patients. Furthermore, the survival analysis of these patients was conducted, which revealed that patients with respiratory dysfunction (**Figure 2B**) (P=0.0037), circulatory dysfunction (P<0.0001) (**Figure 2C**), hematologic dysfunction(P=0.0072) (**Figure 2D**), renal dysfunction(P <0.0001) (**Figure 2E**), and neurological dysfunction (**Figure 2F**) (P<0.0001) had poor survival. However, patients with liver dysfunction survived better(P=0.0354) (**Figure 2G**).

### Characteristics of patients with Post-sepsis persistent organ dysfunction

**Figure 3:**
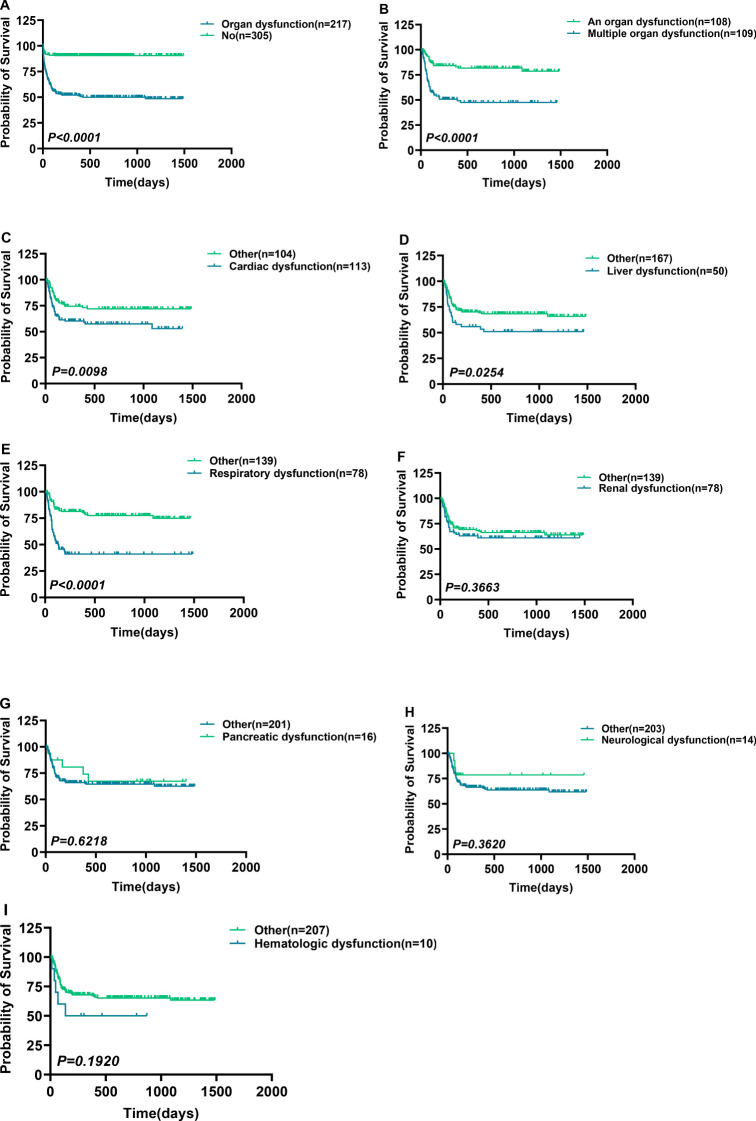
Survival differences in patients with post-sepsis persistent organ dysfunction. Survival differences in patients with post-sepsis persistent organ dysfunction. **(A, B)** Survival comparison of Post-sepsis persistent organ dysfunction patients with patients without Post-sepsis persistent organ dysfunction **(A)**, and patients with post-sepsis persistent multiple organ dysfunction **(B)**. **(C–I)** Ppost-sepsis persistent cardiac **(C)**, liver **(D)**, respiratory **(E)**, renal **(F)**, pancreatic **(G)**, neurological **(H)**, and hematologic **(I)** dysfunction comparison of survival without remaining corresponding dysfunction. *p < 0.05* = statistically difference, and *p > 0.05* = no statistical difference.

This research included 305 patients that indicated no Post-sepsis persistent organ dysfunction and had a mortality rate of 0.33% (1/305), whereas 217 patients had Post-sepsis persistent organ dysfunction (excluding accidental death), 109 indicated post-sepsis multi-organ dysfunction, and 108 patients had Post-sepsis persistent organ dysfunction with a mortality rate of 35.02% (76/217), 50.46% (55/109), and 18.52% (20/108), respectively. The patients were further subdivided based on different organ dysfunction, and **Table 1** shows the basic information of these patients. The survival analysis revealed that the survival rate of patients with one or multiple organ dysfunction was significantly lower than that of patients without Post-sepsis persistent organ dysfunction and those with different organ dysfunction (P<0.0001) (**Figure 3A**, **Supplementary Figures S1A–I**). Furthermore, the survival of patients with Post-sepsis persistent multi-organ dysfunction was also substantially reduced than those with Post-sepsis persistent organ dysfunction (P<0.0001) (**Figure 3B**). Moreover, the survival of patients with Post-sepsis persistent Cardiac dysfunction (P=0.0098) (**Figure 3C**), liver dysfunction (P=0.0254) (**Figure 3D**), and respiratory dysfunctions(P<0.0001) (**Figure 3E**) was worse than other patients with Post-sepsis persistent organ dysfunction. In addition, there was no statistical difference in the survival rate of patients with post-sepsis renal dysfunction (P=0.3664) (**Figure 3F**), pancreatic dysfunction (P=0.6218) (**Figure 3G**), neurological dysfunction (P=0.3620) (**Figure 3H**), hematologic dysfunction (P=0.1920) (**Figure 3I**) than other patients with Post-sepsis persistent organ dysfunction. Overall, it was revealed that patients with Post-sepsis persistent cardiac, liver, and respiratory dysfunctions have poor survival.

**Table 1:** Characteristics of patients with post-sepsis different organ dysfunction.

	Male/Female	Age (years)	SOFA (Mean ± SD)	Mortality (%)	MST ± SE (days)	95%CI
No organ dysfunction (n=305)	203/102	49 ± 17	5 ± 3	0.33(1/305)	585 ± 16.38	552.90-617.10
Organ dysfunction (n=217)	155/62	57 ± 15	6 ± 2	35.02(76/217)	730 ± 55.06	622.08-837.92
Multiple organ disorders (n=109)	83/26	56 ± 15	7 ± 2	50.46(55/109)	565 ± 71.76	424.35-705.65
One organ dysfunction (n=108)	71/37	57 ± 16	6 ± 2	18.52(20/108)	872 ± 108.89	658.57-1085.43
Liver dysfunction (n=50)	35/15	50 ± 14	6 ± 2	48.00(24/50)	966 ± 172.31	628.28-1303.72
Respiratory dysfunction (n=78)	51/27	61 ± 13	7 ± 2	57.69(45/78)	545 ± 249.61	55.76-1034.24
Neurological dysfunction (n=14)	13/31	60 ± 13	7 ± 2	21.43(3/14)	164 ± 291.76	0.00-735.84
Renal dysfunction (n=78)	62/16	53 ± 15	7 ± 2	39.74(31/78)	579 ± 55.03	471.14-686.87
Cardiac dysfunction (n=114)	87/27	59 ± 15	7 ± 2	42.11(48/114)	567 ± 91.00	388.65-745.36
Hematology dysfunction (n=10)	7/3	58 ± 9	7 ± 2	50.00(5/10)	466 ± 177.46	118.17-813.83
Pancreatic dysfunction (n=16)	11/6	43 ± 20	4 ± 2	31.25(5/16)	1034 ± 21.11	992.62-1075.38

### Frequency of pathogens detection in patients with different organ dysfunction

**Figure 4:**
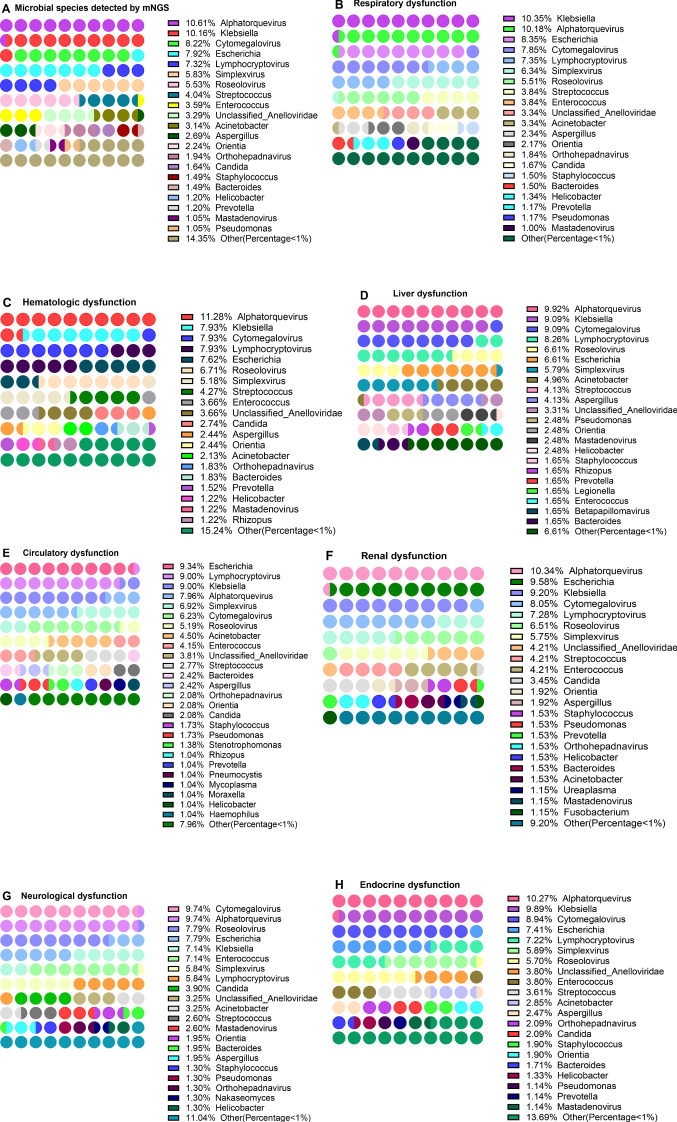
Distribution of pathogen in patients with different organ dysfunction. Distribution of pathogen in patients with different organ dysfunction. **(A)** Pathogen detected by mNGS, **(B–H)** respiratory dysfunction **(B)**, hematologic dysfunction **(C)**, liver dysfunction **(D)**, circulatory dysfunction **(E)**, renal dysfunction **(F)**, neurological dysfunction **(G)**, and Endocrine dysfunction **(H)**.

To explore the distribution of pathogens in sepsis patients with different organ dysfunctions, the genus of 332 mNGS-tested patients was analyzed. Of these, 294 (88.55%) patients were positive and 38 patients were negative for pathogens infection. Among these patients, 58 different pathogens genera were detected, with a total of 669 pathogens species identified across these genera. The first three pathogens with the highest abundance were *Alphatorquevirus* (10.61%), *Klebsiella* (10.16%), and *Cytomegalovirus* (8.22%) (**Figure 4A**, **Supplementary Table S1**). Then the patients who had the NGS test were grouped according to the SOFA 3.0 score table, which revealed that the abundance of *Klebsiella* (10.35%), *Alphatorquevirus* (10.18%), and *Escherichia* (8.35%) was the highest in patients with respiratory dysfunction (**Figure 4B**, **Supplementary Table S1**), while that of *Alphatorquevirus* (11.25%), *Lymphocryptovirus* (7.90%), *Klebsiella* (7.90%), *Cytomegalovirus* (7.90%) and *Escherichia* (7.60%) was highest in patients with Hematologic dysfunction (**Figure 4C**, **Supplementary Table S1**). Furthermore, the detection frequency of *Alphatorquevirus* (9.92%), *Klebsiella* (9.09%), *Cytomegalovirus* (9.09%), and *Lymphocryptovirus* (8.26%) was greatest in patients with liver dysfunction (**Figure 4D**, **Supplementary Table S1**). Moreover, *Escherichia* (9.34%), *Lymphocryptovirus* (9.00%), *Klebsiella* (9.00%), and *Alphatorquevirus* (7.96%) were detected most in patients with circulatory dysfunction (**Figure 4E**, **Supplementary Table S1**). Among patients with renal dysfunction, *Alphatorquevirus* (10.34%), *Escherichia* (9.58%) and *Klebsiella* (9.20%) had the highest detection frequency (**Figure 4F**, **Supplementary Table S1**). Whereas in neurological dysfunction patients, *Cytomegalovirus* (9.74%), *Alphatorquevirus* (9.74%), *Roseolovirus* (7.79%), *Escherichia* (7.79%), *Klebsiella* (7.14%) and *Enterococcus* (7.14%) had the highest abundance (**Figure 4G**, **Supplementary Table S1**). In addition, *Alphatorquevirus* (10.27%), *Klebsiella* (9.89%) and *Cytomegalovirus* (8.94%) had the highest detection frequency among patients with endocrine system dysfunction (**Figure 4H**, **Supplementary Table S1**). These results indicated that infectious pathogens distribution in sepsis patients with different organ dysfunctions is different.

### Frequency of detection of pathogens in patients with Post-sepsis persistent organ dysfunction

**Figure 5:**
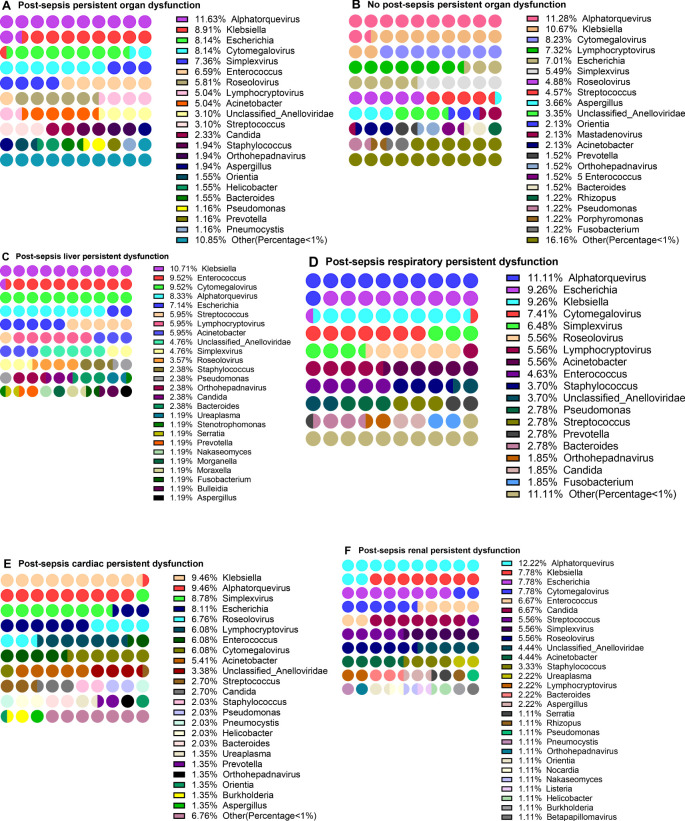
Distribution of pathogens in patients with post-sepsis persistent organ function. Distribution of pathogens in patients with post-sepsis persistent organ function. **(A–F)** Pathogensl distribution of patients with Post-sepsis persistent organ dysfunction **(A)**, no Post-sepsis persistent organ dysfunction **(B)**, post-sepsis persistent liver dysfunction **(C)**, post-sepsis persistent respiratory dysfunction **(D)**, post-sepsis persistent Cardiac dysfunction **(E)**, and post-sepsis persistent renal dysfunction **(F)**.

In addition to different organ dysfunction caused by sepsis, patients often suffer from certain organ dysfunction even after sepsis treatment (here referred to as Post-sepsis persistent organ dysfunction). To explore the distribution of pathogens in patients with Post-sepsis persistent organ dysfunction, 43 patients who died during hospitalization were removed, and the remaining 251 were analyzed. Of these, 101 patients were analyzed for the detection frequency of pathogens. Among the patients with Post-sepsis persistent organ dysfunction, 258 of 44 pathogens were detected. The first three genera with the highest detection frequency were *Alphatorquevirus* (11.63), *Klebsiella* (8.91%), *Escherichia* (8.14%), and *Cytomegalovirus* (8.14%) (**Figure 5A**, **Supplementary Table S2**). There were 150 patients without Post-sepsis persistent organ dysfunction, and indicated 53 of 328 pathogens. The first three pathogens with the highest detection frequency are *Alphatorquevirus* (11.28%), *Klebsiella* (10.67%), and *Cytomegalovirus* (8.23%) (**Figure 5B**, **Supplementary Table S2**).

Furthermore, the patients with post-sepsis persistent different organ dysfunction were further grouped and revealed that in the post-sepsis persistent liver dysfunction patients, *Klebsiella* (10.71%), *Enterococcus* (9.52%), *Cytomegalovirus* (9.52%) and *Alphatorquevirus* (8.33%) had the highest detection frequency (**Figure 5C**, **Supplementary Table S2**). In patients with post-sepsis persistent respiratory dysfunction, the highest detection frequency was of *Alphatorquevirus* (11.11%), *Escherichia* (9.26%), *Klebsiella* (9.26%) *Cytomegalovirus* (7.41%) (**Figure 5D**, **Supplementary Table S2**).In patients with Post-sepsis persistent cardiac dysfunction, *Klebsiella* (9.46%), *Alphatorquevirus* (9.46%), *Simplexvirus* (8.78%), and *Escherichia* (8.11%) had the highest detection frequency (**Figure 5E**, **Supplementary Table S2**). Moreover, *Alphatorquevirus* (12.22%), *Escherichia* (7.78%), *Klebsiella* (7.78%), *Cytomegalovirus* (7.78%), *Enterococcus* (6.67%) and *Candida* (6.67%) had the highest the detection frequency in patients with Post-sepsis persistent renal dysfunction (**Figure 4F**, **Supplementary Table S2**). These results suggest that the distribution of pathogens is not the same in patients with Post-sepsis persistent organ dysfunction.

### Differences in pathogens in patients with organ dysfunction

The aforementioned data indicated that the detection frequency of pathogens in patients with different organ dysfunction is different from the infection rate of the population, and there were also differences in survival rate (**Supplementary Table S1**, **Figure 2**). Therefore, it was suspected that different pathogens infections are related to differential organ dysfunction and survival rates. To confirm this, A chi-square test was performed on patients with different organ dysfunction, the results showed (**Supplementary Table S3**) that the frequency of detection and population infection of *Legionella(P=0.0234)* and *Betapapillomavirus*(P=0.0234) in patients with liver dysfunction was higher than in patients without liver dysfunction. In addition, compared with patients without respiratory dysfunction, patients with respiratory dysfunction have lower detection rates of Haemophilus influenzae (P=0.0303) and population infection rates (P=0.0264). Among patients with neurological dysfunction, the detection frequency and population infection of Mastadenovirus, *Enterococcus*, and *Candida* were significantly higher than in non-neurological disorder patients (P<0.0500). However, infection with Enterococcus(P=0.3200) and Candida(P=0.9189) in patients with neurological dysfunction does not seem to affect survival (**Supplementary Figures S2A, B**). The detection frequency of *Candida* and the incidence of infection was significantly higher in kidney dysfunction and Hematologic dysfunction patients than those without these dysfunction (P<0.0500), but whether infection with *Candida* affects the survival of patients with renal dysfunction does not seem to affect survival (P=0.3115) (**Supplementary Figure S2C**). Furthermore, the detection frequency of *Burkholderia* in Hematological dysfunction patients was lower than that in patients without Hematological dysfunction (P=0.0485), and the infection rate in the population differed significantly (P=0.0500). Among patients with circulatory dysfunction, *Moraxella*’s detection frequency and infection incidence were significantly higher than those without circulatory dysfunction (P<0.0500). In addition, the *Alphatorque* virus infection incidence rate in patients without circulatory system dysfunction was markedly higher than in patients with circulatory system dysfunction (P=0.0476), but the *Alphatorquevirus* infection effect on the survival of patients with circulatory dysfunction does not seem to affect survival (P=0.2636) (**Supplementary Figure S2D**). The genus without survival analysis is due to the sample size of < 5 (**Supplementary Table S1**). Overall, sepsis patients with different organ dysfunction may be infected with different pathogens.

### Differences in pathogens in patients with Post-sepsis persistent organ dysfunction

As mentioned above, in addition to different organ dysfunction caused by sepsis, patients often suffer from certain organ dysfunction even after sepsis treatment. Patients with Post-sepsis persistent organ dysfunction often have poor survival (**Figures 3A–I**). The analysis of post-sepsis dysfunction of different organs revealed that the detection frequency of different pathogens is different in post-sepsis different organ dysfunction patients (**Figure 5**, **Supplementary Table S2**). To explore whether the infection of different pathogens is related to Post-sepsis persistent persistent organ dysfunction, A Chi-square test (**Table 2**) was performed to compare the pathogens between Post-sepsis persistent organ dysfunction patients and those without Post-sepsis persistent organ dysfunction patients. It was found that among patients with Post-sepsis persistent organ dysfunction, the detection frequency and population infection rate of *Enterococcus* was significantly higher than that of patients without Post-sepsis persistent organ dysfunction, but the detection frequency and population infection rate of *Mastadenovirus* were higher in patients without Post-sepsis persistent organ dysfunction. In addition, *Pneumocystis*(P=0.0337) and *Acinetobacter*(P=0.0186) had a higher population infection rate in patients with Post-sepsis persistent organ dysfunction, but there is no statistical difference in detection frequency (P>0.0500). Moreover, no statistical difference was observed in the survival of *Enterococcus*(P=0.9646) and *Acinetobacter-infected*(P*=*0.1184) patients with Post-sepsis persistent organ dysfunction (P>0.0500) (**Supplementary Figures S3A, B**). Among patients with post-sepsis persistent liver dysfunction, the detection frequency and infection rate of *Enterococcus* and *Morganella* were much higher than that of patients without post-sepsis persistent liver dysfunction (P<0.0500). The Population infection rate of *Acinetobacter* was much higher than the patients without post-sepsis persistent liver dysfunction (P = 0.0050), but there is no statistical difference in the detection frequency of *Acinetobacter* in the two groups (P=0.0633). There seems to be no impact on the survival of patients with post-sepsis persistent liver dysfunction infected with *Enterococcus*(P=0.6112) or *Acinetobacter*(P=0.5613)(**Supplementary Figure S3C, D**). Furthermore, the detection frequency and population infection rate of *Staphylococcus* were higher in patients with post-sepsis persistent respiratory dysfunction (P<0.0500), but there was no statistical difference in the detection frequency of *Enterococcus*(P=0.0615) and *Acinetobacter*(P=0.0698). In addition, *Enterococcus-infected* patients with Post-sepsis persistent respiratory dysfunction had worse survival than patients who were not infected with *Enterococcus* (P = 0.0041) (**Supplementary Figure S3E**), Infection with *Acinetobacter* also had no statistical significance (P=0.1162) (**Supplementary Figure S3F**). Moreover, *Enterococcus* and *Candida* have higher detection frequency and population infection rates in patients with post-sepsis persistent Renal dysfunction(P<0.0500). In addition, the infection rate of *Pneumocystis*(P=0.0352), *Staphylococcus*(P=0.0431), and *Listeria(P=0.0352)* was significantly higher in post-sepsis persistent renal dysfunction patients than those without post-sepsis persistent renal dysfunction; however, there was no statistical difference in detection frequency (*Pneumocystis*, P=0.0560*; Staphylococcus*, P=0.0875*; Listeria*,P=0.0560). Furthermore, there was also no statistical difference in the survival rate of *Enterococcus*(P=0.2684) and *Candida-infected*(P=0.3659) patients with post-sepsis persistent renal dysfunction (**Supplementary Figures S3G, H**). *Enterococcus* and *Pneumocystis* also had higher detection frequency and population infection rates in patients with post-sepsis persistent cardiac dysfunction (P<0.0500). Additionally, compared with patients with post-sepsis persistent cardiac dysfunction,the infection rate of *Acinetobacter* was markedly higher than that of patients without post-sepsis persistent cardiac dysfunction (P*=*0.0250), but there was no statistical difference in detection frequency (P*=*0.0586). No statistical difference was observed in the survival of *Enterococcus*(P=0.7821) and *Acinetobacter-infected*(P=0.4701) patients with post-sepsis persistent cardiac dysfunction(**Supplementary Figures S3I-J**). The pathogens without survival analysis are due to the sample size of < 5 (**Supplementary Table S2**).In summary, these results indicate that the types of pathogen infections in patients with different post-sepsis persistent organ dysfunction are different.In addition, patients with post-sepsis persistent respiratory dysfunction infected with *Enterococcus* have worse survival.

**Table 2:** Differences between pathogens and population infections in patients with Post-sepsis persistent organ dysfunction.

	Mastadenovirus		Enterococcus		Candida		Pneumocystis		Acinetobacter		Staphylococcus		Morganella		Listeria	
	A	B	A	B	A	B	A	B	A	B	A	B	A	B	A	B
No organ dysfunction	2.13%	4.67%	1.52%	3.33%	0.91%	2.00%	0.00%	0.00%	2.13%	4.67%	0.91%	2.00%	0.00%	0.00%	0.00%	0.00%
Organ dysfunction	0.00%	0.00%	6.59%	16.83%			1.16%	2.97%	5.04%	12.87%						
χ^2^	5.57	4.85	10.25	13.75			3.83	4.51	3.7	5.54						
P	0.0182	0.0277	0.0014	0.0002			0.0502	0.0337	0.0545	0.0186						
Liver dysfunction			9.52%	32.00%					5.95%	20.00%			1.19%	4.00%		
χ^2^			14	25.61					3.45	7.89			3.91	6.03		
P			0.0002	0.0000					0.0633	0.0050			0.0479	0.0140		
Respiratory dysfunction			4.63%	12.20%					5.56%	14.63%	3.70%	9.76%				
χ^2^			3.5	5.1					3.29	5.04	4	5.49				
P			0.0615	0.0240					0.0698	0.0247	0.0455	0.0192				
Renal dysfunction			6.67%	17.65%	6.67%	17.65%	1.11%	2.94%			3.33%	8.82%			1.11%	2.94%
χ^2^			7.29	10.1	11.09	14.59	3.65	4.44			2.92	4.09			3.65	4.44
P			0.0069	0.0015	0.0009	0.0001	0.0560	0.0352			0.0875	0.0431			0.0560	0.0352
Cardiac dysfunction			6.08%	15.25%			2.03%	5.08%	5.41%	13.56%						
χ^2^			7.42	9.63			6.69	7.74	3.58	5.03						
P			0.0065	0.0019			0.0097	0.0054	0.0586	0.0250						

The differences between the two groups are checked by the card method. The result only retains the difference. *p < 0.05* = statistically difference, and *p > 0.05* = no statistical difference. A: Detection frequency, B: Population infection rate.

### Differences in pathogens of surviving and dead patients

**Figure 6:**
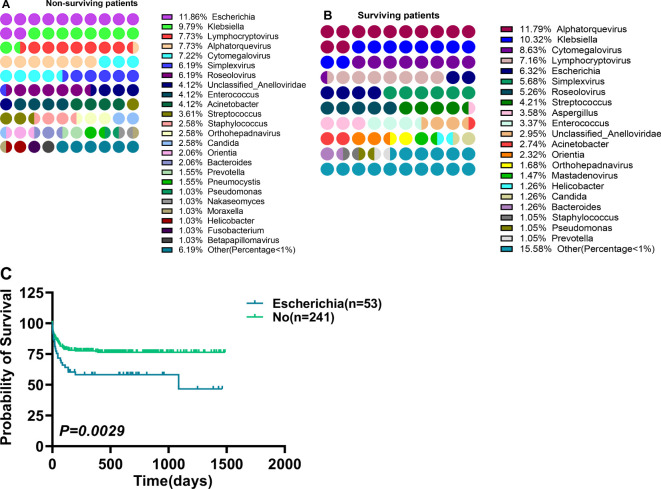
Differences between pathogens distribution in surviving and non-surviving patients, and the survival of patients infected with different pathogens. **(A, B)** Differences between pathogens distribution in surviving **(A)** and non-surviving patients **(B)**, **(C)** Survival differences in patients infected with *Escherichia*. *p < 0.05* = statistically difference, and *p > 0.05* = no statistical difference.

The above data proved that patients with multiple organ dysfunction and post-sepsis dysfunction of different organs are related to different pathogens genera. Whether the pathogens genus was related to patient survival was also assessed. Of 294 patients, 82 died and 212 survived. A total of 475 pathogens types were detected in surviving patients. The first three pathogens with the highest detection frequency were *Alphatorquevirus* (11.79%), *Klebsiella* (10.32%), and *Cytomegalovirus* (8.63%) (**Figure 6A**), the infection rate in the population was 26.42%, 23.11% and 19.34% respectively. Furthermore, 36 of 194 pathogens types were detected in the dead patients, where *Escherichia* (11.86%), *Klebsiella* (9.79%), *Lymphocryptovirus* (7.73%) and *Alphatorquevirus* (7.73%) (**Figure 6B**) were the most abundant pathogens with the infection rate of 28.05%, 23.17%, 18.29%, and 18.29%, respectively. It was found that *Escherichia* had a higher detection frequency(P=0.0161) and population infection rate(P=0.0054) among dead patients (**Table 3**), and alive patients infected with *Escherichia* had a higher mortality rate (P= 0.0029) (**Figure 6C**). Overall, sepsis patients should be carefully monitored for *Escherichia* infection. And take proactive measures against infected patients.

**Table 3:** Differences between surviving and non-surviving patients.

	Escherichia		Aspergillus	
	Detection frequency	Population infection rate	Detection frequency	Population infection rate
Surviving patients	6.32%	14.15%	3.58%	8.02%
Non-surviving patients	11.86%	28.05%	0.52%	1.22%
χ^2^	5.80	7.73	4.95	4.76
P	0.0161	0.0054	0.0263	0.0292

The differences between the two groups are checked by A chi-square test. The result only retains the difference. *p < 0.05* = statistically difference, and *p > 0.05* = no statistical difference.

## Discussion

The literature has indicated that the tissue and organ damage of sepsis patients is mainly caused by either pathogens, a series of inflammatory reactions, or metabolic abnormalities. Whether other sources of infection cause damage to the function of different organs remains to be determined. This research revealed that the distribution of pathogens in survivors and sepsis patients who died is different, and the survival rate of patients with *Enterococcus* infection combined with post-sepsis persistent respiratory dysfunction is also worse.And patients with Respiratory, Liver, Circulatory, Hematologic, Neurological, and Renal dysfunction had poor survival. And patients with post-sepsis persistent organ dysfunction after sepsis have worse survival rates. In addition, we found the infection rates of *Legionella* and *Betapapillomavirus* were higher in patients with liver dysfunction. The infection rates of *Mastadenovirus*, *Enterococcus*, and *Candida* were higher in patients with neurological dysfunction. The infection rates of *Candida* were higher in patients with renal dysfunction and hematologic dysfunction. The infection rates of *Moraxella* were higher in patients with circulatory dysfunction.The infection rates of *Enterococcus, Pneumocystis, and Acinetobacter* were higher in patients with Post-sepsis cardiac dysfunction.The infection rates of *Enterococcus*, Acinetobacter, and Morganella were higher in patients with Post-sepsis liver dysfunction. The infection rates of *Enterococcus, Acinetobacter*, and *Staphylococcus* were higher in patients with Post-sepsis respiratory dysfunction. The infection rates of *Enterococcus, Candida, Pneumocystis, Staphylococcus*, and *Listeria* were higher in patients with Post-sepsis renal dysfunction.In addition, we found that patients with *Escherichia* infection in sepsis had the lowest survival rate.

With advancing research, the mechanism of organ function damage caused by various pathogens is becoming clearer. The interaction of alpha-hydroxyprotein protein and its cell receptor ADAM10 (inducing rapid platelet aggregation) after *Staphylococcus* infection impures endothelial repair function, and promotes neutrophil inflammation signal transmission to form harmful platelet-neutrophil aggregation in organs. Dynamic microthrombus forms in microcirculation cause multi-organ dysfunction of the liver, kidney, and lungs (11–13). Moreover, it has been observed that in *Enterococcus* fecal infection, the determined cluster disulfide bond-forming protein A (DsbA) is necessary for its virulence, inhibits cardiomyocyte’s inflammatory immune response, and promotes apoptosis as well as necrosis, thus forming cardiac micropathy (14). The fecal *Enterococcus* synthesizes peptidoglycan in the liver cell, indicating that fecal *Enterococcus* replicates in the liver cells. After the liver’s innate immune induction, macrophages and neutrophils almost disappear, eventually leading to the formation of *Enterococcus* lesions in the liver. Moreover, this process is not limited to specific cell types and may also occur in other organs such as it has been observed that the intracellular division of fecal *Enterococcus* can be extended to the kidneys (15). In addition, *Enterococcus* is related to the enzymatic ability to convert tyrosine or levodopamine into dopamine, which affects the dopamine content of the body and the function of the nervous system (16, 17). This study also found an increased rate of *Enterococcus* infection in neurological dysfunction (**Supplementary Table S3**), and post-sepsis persistent liver, respiratory, renal, and cardiac dysfunctions (**Table 2**).

This research identified that the mortality rate of patients infected with *Escherichia* is higher (**Figure 5C**). This may be related to *Escherichia’s* ability to acquire virulence factors that cause diseases (adhesiverin, pod membrane, synthesis and secretion of toxins, *etc.*) and antibiotic resistance, which reduces their sensitivity to certain anti-infective molecules (16, 17). In addition, some studies have shown that *Escherichia* has a highly diverse genetic background and VF gene profile in sepsis patients. Furthermore, the expression of *Escherichia* cnf and blaTEM genes in sepsis patients is related to the disease severity, while the expression of the yuA gene is related to mortality (18, 19). Dispersive *Candida* infection mainly occurs in patients with neutropenia, and human neutrophils are the only immune cells that can prevent yeast’s transformation into filamentous growth. Furthermore, increased levels of *Candida albicans’* transcription response in the whole blood (20–22) and the complement system defects enhance the susceptibility to invasive *Candida albicans* infection (23, 24). Patients with neutropenia invasive candidiasis may need corticosteroid treatment after neutrophil reconstruction to avoid adverse reactions and remnants of excessive inflammation because *Candida* raises neutrophils in the kidneys and produces IL-17 to damage the kidneys (25). Here, *Candida* was observed to be associated with the incidence of nervous system, renal, and respiratory system dysfunctions (**Supplementary Table S3**), as well as post-sepsis renal dysfunction (**Table 2**).

Although the mechanism from infection to organ dysfunction comprises the interaction of complex factors, the different pathways are all related to inflammatory reactions, immune disorders, metabolic abnormalities, *etc.* Currently, the sepsis treatment is primarily focused on antipathogensl treatment and organ protection support. However, the high mortality rate of sepsis patients has been constant for the past 10 years. Furthermore, the increased mortality rate of sepsis might be due to the lack of treatment for host immunity that can reverse the disorder (26). Severe inflammation can benefit from anti-inflammatory treatment; however, for immunosuppression, immunostimulant treatment should be considered. A suitable reference for appropriate immunotherapy strategies can be selected from biomarkers such as absolute count of lymphocytes and HLA-DR expression on monocytes can stimulate recombinant glycosylated human IL-7, recombinant interferon-γ (INF-γ), anti-TNF-α, recombinant granulocyte-macrophage colony (GM-CSF) and anti-PD-1 antibodies stimulation for suitable patient’s treatment (26–29). For example, according to the serum ferritin concentration and the expression of HLA-DR on circulating CD14+ monocytes, some patients with macrophage activation-like syndrome (MALS) (high serum ferritin) receive IL-1Ra treatment (30). Since sepsis often involves multiple pathogen infections, organ injuries, and complex immune responses, a single drug cannot reverse these effects throughout the disease range. Therefore, subgroup classification of patients with similar pathophysiology and clinical stratification is more significant for improving the efficacy of immunomodulatory therapy (26, 31, 32).

Overall, we found differences in the types and proportions of pathogens infected in patients with different organ dysfunction and Post-sepsis persistent organ dysfunction. The combination of Escherichia infection and Enterococcus infection with post-sepsis persistent respiratory dysfunction can affect the survival of patients. We should strengthen the management of sepsis patients, especially those with Post-sepsis persistent organ dysfunction. However, this study has certain limitations: 1) Because there are often multiple pathogens and organ dysfunctions in sepsis patients, a single pathogen or organ dysfunction cannot be studied. This research only indicated their possible relation. 2) This is a single-center data analysis, which may not represent all sepsis patients. These factors are universal and objective and also affect the research of other institutions. The mechanism of organ function damage and legacy organ dysfunction is complex crosstalk, which has not been fully analyzed. The early reaction of sepsis patients is the activation of the body’s self-protective mechanism for survival, and with the disease progression, this self-protection mechanism causes serious damage to the body. Therefore, some treatment strategies should be altered and in addition to the removal of pathogens and supportive treatment of organ function, sepsis management should also be considered. For instance, initially, active empirical antibiotic treatment should be given and a plan should be quickly formulated to identify pathogens for accurate treatment and maximum eradication of pathogens. Furthermore, biomarkers should be monitored in real-time during treatment, and suitable immune stimulation (GM-CSF, IFNγ, IL-7, *etc.*), inflammatory response suppression (corticosteroids, TNF-α antagonists, PD-1 antagonists, IL-1 receptor antagonists) according to biomarkers and the patient’s clinical characteristics (Akinra), metabolic regulation (cholesterol supplementation, iron removal therapy, antioxidant therapy, *etc.*), and nutrient supplementation treatment should be considered to achieve precision. Moreover, after patients are discharged from the hospital, especially those with Post-sepsis persistent organ dysfunction, should receive more attention. If the patient has PICS, it may lead to long-term chronic diseases, re-infection, and other diseases. For such patients, it is recommended to formulate a strategy to promote the elimination of inflammation, antioxidants, immune balance, and metabolic recovery according to the patient’s situation.

## Data Availability

The original contributions presented in the study are included in the article/**Supplementary Material**. Further inquiries can be directed to the corresponding authors.
