# Supplementary material for: The type of pathogen is associated with organ failure and legacy dysfunction in patients with sepsis
Source: Front Immunol. 2025 Mar 31;16:1541634. doi: 10.3389/fimmu.2025.1541634 (PMC11994601; doi:10.3389/fimmu.2025.1541634)
Supplement: Supplementary file 1 [file Table1.docx]

Supplementary Figure 1. Survival differences between patients with different post-sepsis persistent organ dysfunction and patients without post-sepsis persistent organ dysfunction

Note: (A)Post-sepsis persistent one-organ dysfunction, (B) Post-sepsis persistent multiple organ dysfunction, (C) Post-sepsis persistent liver dysfunction, (D) Post-sepsis persistent cardiac dysfunction, (E) Post-sepsis persistent renal dysfunction, (F) Post-sepsis persistent respiratory dysfunction, (G) Post-sepsis persistent pancreatic dysfunction, and (H) Post-sepsis persistent neurological dysfunction. (I) Post-sepsis persistent Hematologic dysfunction Comparison of survival patients without post-sepsis persistent organ dysfunction. *p < 0.05* = statistically difference, and *p > 0.05* = no statistical difference.

Supplementary Figure 2. Survival differences of pathogen patients with different organ dysfunction

Note: A-B: Survival differences between (A) *Enterococcus* and (B) *Candida-infected* patients with neurological dysfunction. C-D: Survival differences between (C) *Candida* and (D) *Alphatorquevirus-infected* patients with kidney dysfunction. The survival analysis only assessed the sample size of ≥ 5. *p < 0.05* = statistically difference, and *p > 0.05* = no statistical difference.

Supplementary Figure 3. Survival differences of pathogen patients with post-sepsis persistent different organ dysfunction

Note: A-B: Survival difference of post-sepsis persistent organ dysfunction patients infected with (A) *Enterococcus* and (B) *Acinetobacter.* C-D: Survival difference of post-sepsis liver dysfunction patients infected with (C) *Enterococcus* and (D) *Acinetobacter.* E-F: Survival difference of post-sepsis respiratory dysfunction in patients infected with (E) *Enterococcus* and (F) *Acinetobacter.* G-H: Survival difference of post-sepsis renal dysfunction in patients infected with (G) *Enterococcus* and (H) *Candida.* I-J: Survival difference of post-sepsis mental dysfunction in patients infected with (I) *Enterococcus and* (J) *Acinetobacter*. The survival analysis only assessed the sample size of ≥ 5. *p < 0.05* = statistically difference, and *p > 0.05* = no statistical difference.
